# Establishment of a Lactylation-Related Gene Signature for Hepatocellular Carcinoma Applying Bulk and Single-Cell RNA Sequencing Analysis

**DOI:** 10.1155/ijog/3547543

**Published:** 2025-02-14

**Authors:** Lianghe Yu, Yan Shi, Zhenyu Zhi, Shuang Li, Wenlong Yu, Yongjie Zhang

**Affiliations:** ^1^Hepatobiliary Surgery, The Third Affiliated Hospital, Naval Military Medical University, Shanghai, China; ^2^Bioinformatics R&D Department, Hangzhou Mugu Technology Co., Ltd, Hangzhou, China

**Keywords:** bulk RNA-seq, gene signature, hepatocellular carcinoma, immune infiltration, immunotherapy, lactylation, scRNA-seq

## Abstract

**Background:** Lactylation is closely involved in cancer progression, but its role in hepatocellular carcinoma (HCC) is unclear. The present work set out to develop a lactylation-related gene (LRG) signature for HCC.

**Methods:** The lactylation score of tumor and normal groups was calculated using the gene set variation analysis (GSVA) package. The single-cell RNA sequencing (scRNA-seq) analysis of HCC was performed in the “Seurat” package. Prognostic LRGs were selected by performing univariate and least absolute shrinkage and selection operator (LASSO) Cox regression analyses to develop and validate a Riskscore model. Functional enrichment analysis was conducted by gene set enrichment analysis (GSEA) using the “clusterProfiler” package. Genomic characteristics between different risk groups were compared, and tumor mutational burden (TMB) was calculated by the “Maftools” package. Immune cell infiltration was assessed by algorithms of cell-type identification by estimating relative subsets of RNA transcript (CIBERSORT), microenvironment cell populations-counter (MCP-counter), estimating the proportions of immune and cancer cells (EPIC), tumor immune estimation resource (TIMER), and single-sample gene set enrichment analysis (ssGSEA). Immunotherapy response was predicted by the tumor immune dysfunction and exclusion (TIDE) algorithm. Drug sensitivity was analyzed using the “pRRophetic” package. A nomogram was established using the “rms” package. The expressions of the prognostic LRGs in HCC cells were verified by in vitro test, and cell counting kit-8 (CCK-8), wound healing, and transwell assays were carried out to measure the viability, migration, and invasion of HCC cells.

**Results:** The lactylation score, which was higher in the tumor group than in the normal group, has been confirmed as an independent factor for the prognostic evaluation in HCC. Six prognostic LRGs, including two protective genes (*FTCD* and *APCS*) and four risk genes (*LGALS3*, *C1orf43*, *TALDO1*, and *CCT5*), were identified to develop a Riskscore model with a strong prognostic prediction performance in HCC. The scRNA-seq analysis revealed that *LGALS3* was largely expressed in myeloid cells, while *APCS*, *FTCD*, *TALDO1*, *CCT5*, and *C1orf43* were mainly expressed in hepatocytes. The high-risk group was primarily enriched in the pathways involved in tumor occurrence and development, with higher T cell infiltration. Moreover, the high-risk group was found to be less responsive to immunotherapy but was more sensitive to chemotherapeutic drugs. By integrating Riskscore and clinical features, a nomogram with a high predictive accuracy was developed. Additionally, *C1orf43*, *CCT5*, *TALDO1*, and *LGALS3* were highly expressed in HCC cells. Silencing *CCT5* inhibited the viability, migration, and invasion of HCC cells.

**Conclusion:** The present work developed a novel LRG gene signature that could be considered a promising therapeutic target and biomarker for HCC.

## 1. Introduction

Hepatocellular carcinoma (HCC) derives from hepatocytes and accounts for 75%–85% of all liver cancer cases [1]. The incidence and mortality of HCC are significantly high, causing around 906,000 new cases and 830,000 deaths in 2020 worldwide [2]. HCC is an invasive malignancy that usually exhibits poor prognostic outcomes, with a 5-year survival of 18% [3, 4]. The development of HCC is a complex process, especially in the presence of cirrhosis [5, 6]. The risk factors of HCC are mainly chronic hepatitis B/C virus infection, genetic susceptibility, environment, alcohol drinking, and smoking [7]. About 80% of patients cannot be timely treated due to inconspicuous clinical symptoms of HCC [8]. At present, HCC are treated by surgical excision, liver transplantation, and radiofrequency ablation [9–11], which are largely effective to early-stage HCC patients [12]. For late-stage HCC patients, radiotherapy, chemotherapy, immunotherapy, and targeted treatment are the standard therapies [13]. Recently, immune checkpoint inhibitors (ICIs) and tyrosine kinase inhibitors have been approved for late-stage HCC treatment [14]. At present, the 5-year survival chance of HCC patients still remains unfavorable due to drug resistance, lower immunotherapy response as well as tumor metastasis and recurrence [15]. Thus, exploring novel and effective signature has a high clinical significance for the early diagnosis, improving prognosis, and treatment intervention for HCC patients.

Disordered metabolism is a manifestation of cancers [16]. Lactic acid, a metabolic product of glycolysis, has multiple biological functions including providing energy, serving as signaling molecules and immunomodulators [17]. Lactylation is a type of posttranslational modification that involves the adherence of lactate to lysine residues and exists in many human tissues [18]. It was reported that lactylation plays a crucial role in tumor metabolic reprogramming [19, 20], polarization of macrophage [21], regulation of gene expression, and cancer progression [22]. Enhanced glycolysis and increased lactate provide a precursor for histone lysine modification, acting as a hallmark of HCC [23]. Yang et al. found abundant protein lactylation in HCC tissues through immunostaining, and they further verified that lactylation at K28 could suppress the function of adenylate kinase 2 to facilitate the growth and metastasis of HCC cells [24]. The lactate accumulation and lactylation in HCC could enhance the immunosuppressive characteristics of the tumor microenvironment (TME), which is a vital factor influencing the development, immunotherapy, and prognosis of HCC [25]. Recently, studies suggested that lactylation-related genes (LRGs) can act as prognostic markers and therapeutic targets for numerous cancers, including prostate cancer [26], pancreatic adenocarcinoma [27], and HCC, but study on the potential functions and prognostic values of LRGs in HCC is limited.

This study used bulk and single-cell RNA sequencing (scRNA-seq) analysis to develop a novel LRG signature to predict the immunotherapy responses and prognostic outcomes for patients suffering from HCC. Three HCC-related datasets were collected from The Cancer Genome Atlas (TCGA) and Gene Expression Omnibus (GEO) databases. Lactylation score was calculated for each sample in The Cancer Genome Atlas–hepatocellular carcinoma (TCGA-HCC), and the scRNA-seq analysis was performed using the GSE112271 dataset. Then, prognostic LRGs were screened to develop a Riskscore model, which was validated in the GSE43619 dataset. We also compared the functional enrichment pathways, genomic characteristics, immune cell infiltration, drug sensitivity, and immunotherapy responses between different risk groups. Furthermore, a nomogram was developed and verified by integrating Riskscore and clinical features. Finally, the mRNA expressions of the key LRGs and their potential biological functions were validated based on an in vitro HCC cell model.

## 2. Material and Methods

### 2.1. Data Collection and Preprocessing

The clinical information, somatic mutation data, and gene expression data of HCC patients were collected from the TCGA database (https://portal.gdc.cancer.gov) [28]. Then, the fragments per kilobase of transcript per million fragments mapped (FPKM)-normalized RNA-seq data in TCGA-HCC were converted to transcripts per million (TPM) format and log2 transformed. A sum of 342 tumors and 50 normal samples with survival time > 30 days were retained. The TCGA-HCC dataset was a training set.

GSE43619 (https://www.ncbi.nlm.nih.gov/geo/query/acc.cgi?acc=GSE43619) dataset containing the gene expression data and clinical information of 88 HCC patients was obtained from the GEO database and utilized as a validation set.

The scRNA-seq data of seven HCC samples in the GSE112271 dataset (https://www.ncbi.nlm.nih.gov/geo/query/acc.cgi?acc=GSE112271) were collected from the GEO database. Firstly, the scRNA-seq data was filtered to ensure that each gene was expressed in 3 cells at least and each cell expressed a minimum of 200 genes. The proportion of rRNA and mitochondria was calculated by the PercentageEigenSet function, and the cells with the percentage of mitochondrial reads higher than the third quartile of samples were removed. Next, the ScaleData function was utilized to normalize the data, and principal component analysis (PCA) dimensionality reduction was performed to determine the anchor points (dim = 20). The batch effects between different samples were removed by “harmony” R package [29].

### 2.2. Lactylation Score Analysis

A total of 332 LRGs were obtained from a previous study [30]. Then, the lactylation score of each sample in TCGA-HCC cohort was calculated by single-sample gene set enrichment analysis (ssGSEA) in gene set variation analysis (GSVA) R package [31]. The samples were assigned by the median value of lactylation score into low- and high-lactylation groups, followed by performing the Kaplan–Meier (K-M) analysis to the overall survival (OS) of patients using “survival” R package [32]. The correlation between the American Joint Committee on Cancer (AJCC) stage, grade, and lactylation score was analyzed. Subsequently, whether lactylation score was an independent prognostic factor for HCC was investigated by subjecting clinical features (AJCC stage, grade, age, and gender) and lactylation score to the univariate and multivariate Cox regression analysis [33].

### 2.3. The scRNA-Seq Analysis

The scRNA-seq analysis was performed based on the GSE112271 dataset using “Seurat” R package [34, 35]. After dimensionality reduction by the uniform manifold approximation and projection (UMAP) analysis, the cells were clustered by the FindNeighbors and FindClusters functions at the resolution (0.1). The marker genes provided by the CellMarker 2.0 database (http://bio-bigdata.hrbmu.edu.cn/CellMarker/) were used to annotate the cell types. In addition, the lactylation activity scores of all cell types were calculated using the “AUCell” R package [36]. All the cells were divided by the median of lactylation activity score into high- and low-LRG groups for subsequent analysis.

### 2.4. Differentially Expressed Gene (DEG) Analysis

The DEGs between different groups were identified through the “limma” R package [37] under the criteria of |log2foldchange(FC)| > 1 and *p*.adj < 0.05 for the tumor and normal groups in TCGA-HCC cohort and under logfc.threshold = 0.25 for high- and low-LRG groups in the GSE112271 dataset. Then, the DEGs of the TCGA-HCC and GSE112271 datasets were intersected to screen the hub genes associated with lactylation in HCC [34].

### 2.5. Construction and Verification of a Riskscore Model

First, the univariate Cox regression analysis (*p* < 0.05) was conducted on the hub genes using “survival” R package [38]. Then, the least absolute shrinkage and selection operator (LASSO) Cox regression analysis in the “glmnet” R package was used to reduce gene numbers [39], and the prognostic LRGs for HCC were identified to construct the Riskscore model. Next, the stepwise regression analysis was employed to calculate the Riskscore for each sample [40].

 $$\begin{array}{c} \text{Riskscore}=\sum \beta i*\text{ExPi} \end{array}$$

*β* represents the gene coefficient in the Cox regression model, and *i* represents the gene expression.

HCC patients were separated by the median of Riskscore into high- and low-risk groups. The OS and progression-free survival (PFS) between different risk groups in the TCGA-HCC cohort were compared by conducting K-M analysis in “survival” R package [41]. The “timeROC” R package was employed to plot the receiver operating characteristic (ROC) curve [42]. Meanwhile, the predictive performance of Riskscore was validated in the GSE43619 dataset.

### 2.6. Functional Enrichment Analysis

Gene Ontology (GO) enrichment analysis was conducted using GSEA in “clusterProfiler” R package [43] to compare the differences in biological process (BP) terms between the groups. Based on the Hallmark pathway gene set collected from the Molecular Signatures Database (MSigDB) (https://www.gsea-msigdb.org/gsea/msigdb/), the Hallmark pathway score was calculated using the “GSVA” R package for different risk groups [44]. The pathway activities of oncogenic signaling pathways in the TCGA-HCC cohort were analyzed using “progeny” R package [45], and the correlation with Riskscore was evaluated.

### 2.7. Genomic Characteristics

Genomic characteristics between the risk groups in the TCGA-HCC cohort were compared. The genomic characteristics of TCGA-HCC samples including tumor mutational burden (TMB), nonsilent mutation rate, number of segments, fraction altered, proliferation, and TGF-*β* response were obtained from a past study [46]. TMB was calculated according to the somatic mutation spectrum of the TCGA-HCC queue using “Maftools” R package [47].

### 2.8. Analysis of Immune Cell Infiltration

The infiltration of immune cells between the risk groups in the TCGA-HCC cohort was evaluated by CIBERSORT [48], microenvironment cell populations-counter (MCP-counter) [49], estimating the proportions of immune and cancer cells (EPIC) [50], and tumor immune estimation resource (TIMER) [51]. Moreover, 28 tumor-infiltrating lymphocyte (TIL) scores were calculated by ssGSEA using “GSVA” R package [52], and the gene set was acquired from the previous literature [53]. The expressions of immunomodulatory-related genes (chemokine, immunoinhibitor, immunostimulator, major histocompatibility complex (MHC), and receptor) [54] in the risk groups were measured.

### 2.9. Assessment of Immunotherapy Responses and Drug Sensitivities

The tumor immune dysfunction and exclusion (TIDE) algorithm was utilized to assess the differences in immunotherapy responses between the risk groups in the TCGA-HCC cohort. Meanwhile, the exclusion score and TIDE score were calculated, with a higher TIDE score showing a greater possibility of immune escape and lower efficacy [55]. The IC50 values of chemotherapeutic drugs were calculated using “pRRophetic” R package to assess the chemotherapeutic drug sensitivity [56]. The relationship between Riskscore and IC50 was further analyzed to identify the drugs with |cor| > 0.5.

### 2.10. Establishment and Verification of a Nomogram

Whether Riskscore was an independent factor for the prognostic evaluation in HCC was assessed by performing univariate and multivariate Cox regression analysis on the clinical features (age, gender, stage, and grade) and Riskscore. Then, a nomogram was created with “rms” R package by integrating Riskscore and clinical features [57]. The prediction accuracy and reliability of the nomogram were reflected by the calibration curve and decision curve analysis (DCA). Furthermore, the relationship between clinical characteristics and Riskscore was examined, and the concordance index (C-index) of the nomogram, Riskscore, and various clinical features was compared.

### 2.11. Cell Lines and Transfection

Transformed Human Liver Epithelial-2 (THLE-2) and HCC cell line Huh-7 were gifts from friends. The cells were cultivated using Dulbecco's modified Eagle's medium (DMEM) added with 1% antibiotic/antifungal solution, 1% glutamine, 10% fetal bovine serum (FBS) at 37°C, and 5% CO_2_. Then, a cell transfection assay was conducted for *CCT5* silencing. Small interfering (si) RNA of *CCT5* (*si-CCT5*, target sequence: 5⁣′-CTGTAGCAAATACAATGAGAACA-3⁣′) and small interfering negative control (si-NC) were purchased from Sangon Biotech (Shanghai, China). Lipofectamine 2000 (Invitrogen) was applied to cell transfection for 48 h at 37°C following the instructions.

### 2.12. Quantitative Real-Time Polymerase Chain Reaction (qRT-PCR)

Firstly, total RNA was separated from THLE-2 and Huh-7 cells with Trizol reagent (B610409-0100, Sangon, China) and reverse-transcribed to acquire cDNA utilizing a cDNA Synthesis Kit (KR107-02, Tiangen, China). Then, to determine the expression levels of prognostic LRGs, qRT-PCR was conducted using the FastReal qPCR SYBR Green PreMix (FP217-02, Tiangen, China) following the instructions. Amplification was conducted at 95°C for 5 min, 40 cycles at 95°C for 15 s, and 58°C for 30 s. The 2^−ΔΔCT^ method was applied to calculate the relative expressions for the prognostic LRGs, with *GAPDH* as an internal control [58, 59]. See Supporting Information 2: Table S1 for the primer sequences of this study.

### 2.13. Western Blot

Lysates from Huh-7 cells were isolated using a radioimmunoprecipitation assay buffer including 0.01% of a freshly prepared protease inhibitor cocktail (Sigma). Equal quantities of proteins were then isolated via Sodium Dodecyl Sulfate–Polyacrylamide Gel Electrophoresis (SDS-PAGE) and moved onto nitrocellulose membranes (Millipore, Bedford, United States), which were incubated with primary antibodies targeting *CCT5* and *GAPDH* at 4°C for 12 h. Next, appropriate horseradish–peroxidase–conjugated secondary antibodies were incubated with the membranes. The blots were detected employing an enhanced chemiluminescence system (Bio-Rad, Richmond, California, United States).

### 2.14. Cell Viability Test

Cell counting kit-8 (CCK-8) test was performed to analyze the effect of *CCT5* silencing on the viability of HCC cells Huh-7 [60]. Briefly, Huh-7 cells at a concentration of 1 × 10^4^ cells/well were planted into 96-well plates. Afterwards, 10 *μ*L CCK-8 solution (E-CK-A362, Elabscience, China) was supplemented into each well at 37°C for 1 h. The absorbance in each well at 450 nm was detected by a microplate reader (Multiskan SkyHigh, Thermo Fisher, United States).

### 2.15. Wound Healing Assay

The impact of *CCT5* silencing on the migratory capability of Huh-7 cells was measured by wound healing assay [61]. First, the Huh-7 cells (1 × 10^4^ cells/well) were cultivated into a 6-well plate for overnight, and then, a wound was scratched using a sterile 20 *μ*L pipette. The cells were incubated at 37°C for 48 h in a serum-deleted medium. A DM IL LED inverted microscope (Leica, Germany) was applied to capture the representative picture, and the wound closure (percent) of Huh-7 cells was calculated using the ImageJ software.

### 2.16. Transwell Assay

The effect of *CCT5* silencing on the invasion of Huh-7 cells was measured through transwell assay [62]. Briefly, the transfected Huh-7 cells (1 × 10^4^ cells/well) were inoculated into the upper transwell chamber (8 *μ*m, FTW010-24Ins, Beyotime, China) with 200 *μ*L serum-free medium. The diluted Matrigel (C0372, Beyotime, China) was precoated on the Transwell chamber. The lower chamber was supplemented with 650 *μ*L DMEM comprising 10% FBS (CBP60202M, Cobioer, China). After 24 h, the Huh-7 cells invaded in the lower chamber were fixed by 4% paraformaldehyde for 30 min and dyed with 0.1% crystal violet for 15 min. The invasive Huh-7 cells were quantified manually under an inverted microscope (DM IL LED, Leica, Germany).

### 2.17. Statistical Analysis

Data from bioinformatics analyses were analyzed in R language (Version 3.6.0). Wilcoxon rank-sum test was used to calculate the differences between two continuous variable groups. Correlation analysis was conducted with the Spearman method. Survival differences of patients between different groups were analyzed by applying a log-rank test. SPSS 19.0 was used in all the statistical analyses, and the data were presented with mean ± standard deviation. Statistically significant was defined when *p* < 0.05.

## 3. Results

### 3.1. Lactylation Score Was an Independent Factor for Predicting the Prognostic Outcomes of HCC

**Figure 1:**
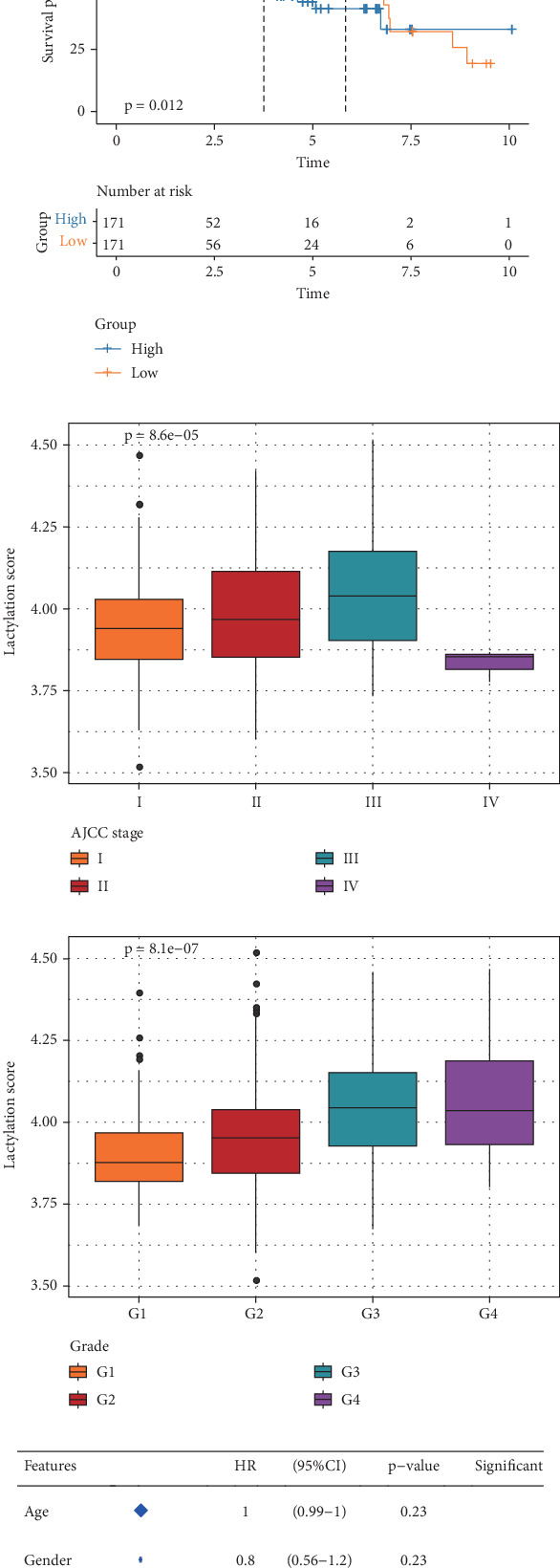
The lactylation score analysis in the TCGA-HCC cohort. (a) Lactylation score in tumor and normal groups. (b) Kaplan–Meier (K-M) curve for overall survival (OS) between high- and low-lactylation groups. (c) Relationship between lactylation score and AJCC stage progression. (d) Correlation between lactylation score and grade progression. (e, f) Univariate and multivariate Cox regression analysis of the lactylation score and clinical features. ⁣^∗∗∗∗^*p* < 0.0001; ⁣^∗∗∗^*p* < 0.001; ⁣^∗∗^*p* < 0.01.

The results from ssGSEA showed that the lactylation score of the tumor group was notably higher in comparison to normal group in the TCGA-HCC cohort (Figure 1(a)). According to the K-M curve, the high-lactylation group exhibited a lower survival probability and a worse prognostic outcome than the low-lactylation group (Figure 1(b)). Meanwhile, the lactylation score increased with the development of grades (G1, G2, G3, and G4) and clinical AJCC stages (I, II, and III) (Figures 1(c) and 1(d)), pointing to the correlation between lactylation score and the degree of malignancy of HCC. The hazard ratio (HR) value of the lactylation score was 9.8, and the 95% confidence interval (CI) was 3.3–29, as shown by the univariate Cox regression analysis (Figure 1(e)). In multivariate Cox regression analysis, the HR value of the lactylation score was 7.7, and the 95% CI was 2.3–26 (Figure 1(f)). These findings suggested that the lactylation score was an independent factor for the prognosis of HCC.

### 3.2. The scRNA-Seq Analysis of HCC Was Performed

**Figure 2:**
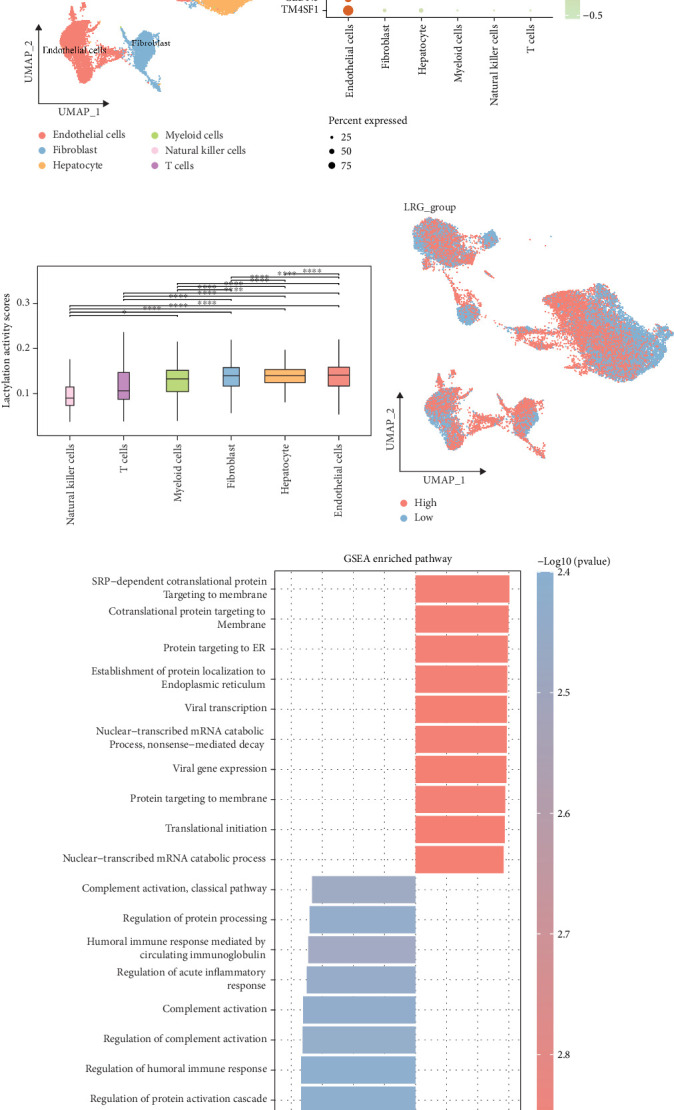
The scRNA-seq analysis on the GSE112271 dataset. (a) UMAP plot of different cell types in HCC. (b) Bubble diagram of marker genes in each cell type. (c) Lactylation activity scores of different cell types. ⁣^∗∗∗∗^*p* < 0.0001; ⁣^∗^*p* < 0.05. (d) UMAP plot of AUCell of lactylation activity in each cell. (e) Difference in GO_BP between high- and low-LRG groups.

Next, we further analyzed the functional properties of LRGs in specific cell types of HCC and their impact on the tumor progression. The scRNA-seq analysis of HCC in the GSE112271 dataset revealed six cell types (Figure 2(a)), namely, endothelial cells (marked with *TM4SF1*, *CLDN5*, *PLVAP*, and *VWF*), fibroblasts (marked with *ACTA2* and *TAGLN*), hepatocytes (marked with *CYP2E1* and *APOA2*), myeloid cells (marked with *C1QB*, *C1QA*, and *AIF1*), natural killer cells (marked with *CCL5*, *GNLY*, *NKG7*, *IFNG*, and *GZMA*), and T cells (marked with *LTB*, *CD3D*, and *CCL5*) (Figure 2(b)). Fibroblasts, hepatocytes, and endothelial cells exhibited higher lactylation activity scores (Figure 2(c)). All the cells were divided by the median of lactylation activity score into high- and low-LRG groups (Figure 2(d)). GSEA revealed that the high-LRG group was primarily enriched in nuclear-transcribed mRNA catabolic process, protein localization to the endoplasmic reticulum, and establishment of cotranslational protein targeting to membrane, while the low-LRG group was principally enriched in protein activation cascade, regulation of acute inflammatory response, regulation of humoral immune response, etc. (Figure 2(e)).

### 3.3. Riskscore Model Was Constructed Based on the Six Prognostic LRGs in HCC

**Figure 3:**
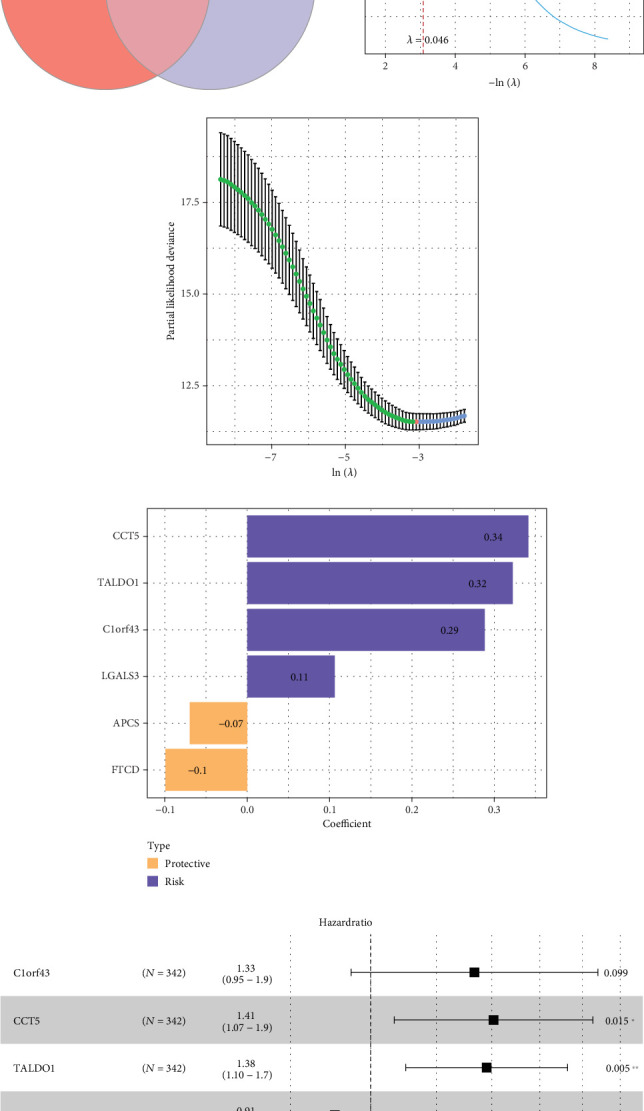
Construction of the Riskscore model. (a) Venn diagram of DEGs in TCGA-HCC and DEGs between high- and low-LRG groups. (b, c) Results of reducing the gene range by LASSO Cox regression analysis. (d) Coefficients of model genes. (e) Multivariate forest plot of model genes. ⁣^∗∗^*p* < 0.01; ⁣^∗^*p* < 0.05.

A total of 2775 DEGs were screened between normal and tumor groups in the TCGA-HCC cohort, and 345 DEGs were identified between high- and low-LRG groups in the GSE112271 dataset. The intersection of these two sets contained 132 hub genes associated with lactylation in HCC (Figure 3(a)). By LASSO Cox regression analysis, the gene numbers were reduced to a suitable range to construct the optimal model when the lambda = 0.046 (Figures 3(b) and 3(c)). Then, six prognostic LRGs incorporating two protective genes (*FTCD* and *APCS*) and four risk genes (*LGALS3*, *C1orf43*, *TALDO1*, and *CCT5*) were screened and utilized to establish a Riskscore model: Riskscore = 0.288∗C1orf43 + 0.341∗CCT5 + 0.322∗TALDO1 + (−0.099∗FTCD) + 0.106∗LGALS3 + (−0.07∗APCS) (Figures 3(d) and 3(e)).

### 3.4. Riskscore Model Exhibited a Strong Prognostic Prediction Performance in HCC

**Figure 4:**
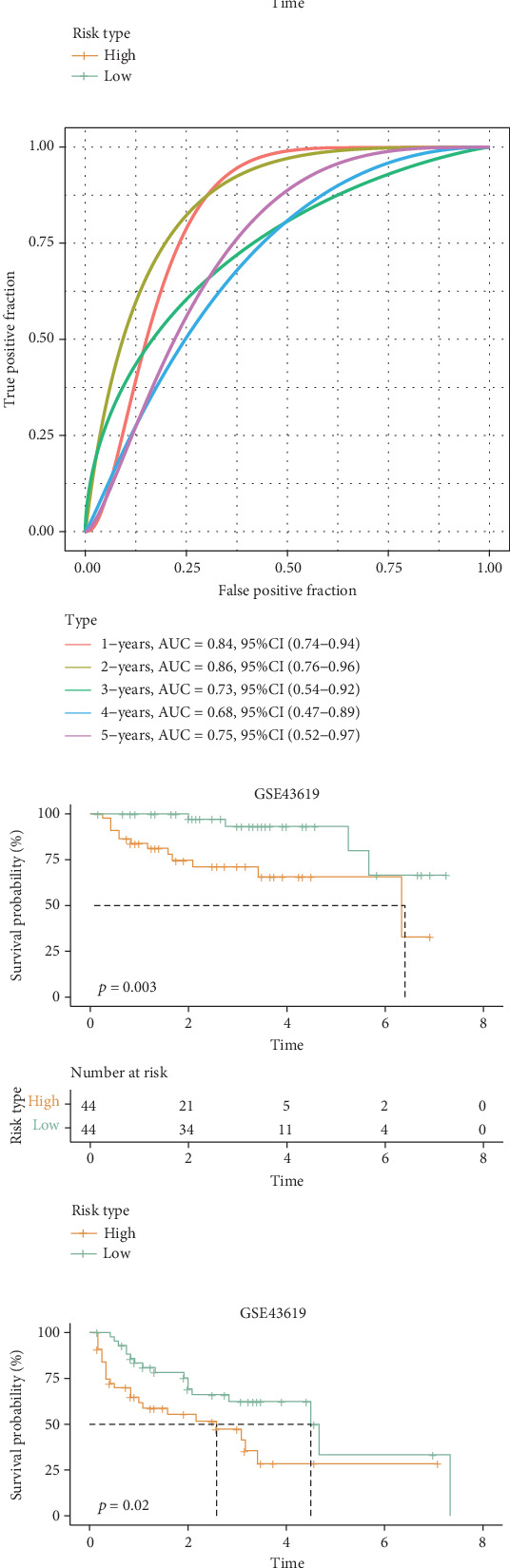
Validation of the Riskscore model. (a) ROC curve of the Riskscore model for 1–5 years in the TCGA-HCC cohort. (b, c) K-M curves for OS and PFS between high- and low-risk groups in the TCGA-HCC cohort. (d) ROC curve of the Riskscore model for 1–5 years in the GSE43619 dataset. (e, f) K-M curves for OS and progression-free survival (PFS) between high- and low-risk groups in the GSE43619 dataset. (g, h) Expression heatmaps of model genes in the TCGA-HCC cohort and GSE43619 dataset. (i) Expression bubble plot of model genes in the scRNA-seq dataset GSE112271.

The HCC patients were assigned into low- and high-risk groups by the median of the Riskscore. The area under ROC curve (AUC) showed that the Riskscore model was accurate with a 1-year AUC of 0.78, 2-year AUC of 0.73, 3-year AUC of 0.72, 4-year AUC of 0.72, and 5-year AUC of 0.72 in TCGA-HCC cohort (Figure 4(a)). K-M curves of OS and PFS demonstrated that in the TCGA-HCC cohort, the high-risk group showed a lower survival probability and worse prognostic outcomes than the low-risk group (Figures 4(b) and 4(c)). Moreover, the predictive performance of the Riskscore model was also verified in the GSE43619 dataset, which showed similar results to the TCGA-HCC cohort (Figures 4(d), 4(e), and 4(f)). Based on the above data, the Riskscore model has manifested a strong prognostic prediction performance in HCC. Furthermore, the expressions of six prognostic LRGs between the two risk groups were compared in TCGA-HCC and GSE43619 datasets. It was found that *APCS* and *FTCD* were high-expressed in the low-risk group, while *C1orf43*, *CCT5*, *TALDO1*, and *LGALS3* were high-expressed in the high-risk group (Figures 4(g) and 4(h)). In the scRNA-seq dataset GSE112271, the expression of *LGALS3* was mainly expressed in myeloid cells, while *APCS*, *FTCD*, *TALDO1*, *CCT5*, and *C1orf43* were mainly expressed in hepatocytes (Figure 4(i)).

### 3.5. Functional Enrichment Analysis and Genomic Characteristics

**Figure 5:**
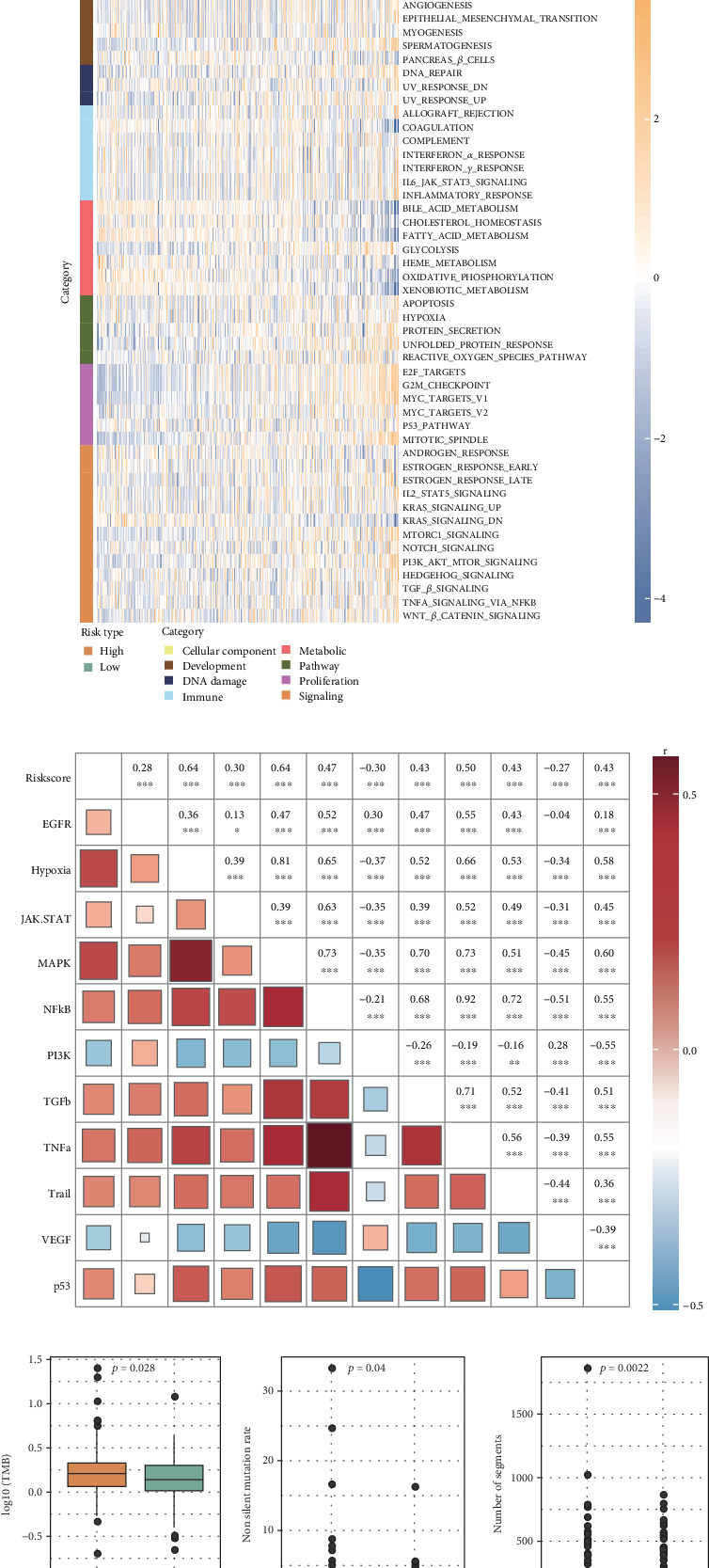
Functional enrichment analysis in the TCGA-HCC cohort. (a) GO_BP in the high-risk group by GSEA. (b) Difference in Hallmark pathway activity between high- and low-risk groups. (c) Correlation between Riskscore and oncogenic signaling pathway activities. ⁣^∗∗∗^*p* < 0.001; ⁣^∗∗^*p* < 0.01; ⁣^∗^*p* < 0.05. (d) Differences in genomic characteristics between different risk groups.

GSEA revealed that the high-risk group was notably enriched in Meiotic Cell Cycle Phase Transition, Mitotic Spindle Elongation, and Spindle Elongation (Figure 5(a)). The Hallmark pathway score related to tumor occurrence and development was higher in the high-risk group, such as epithelial–mesenchymal transition, angiogenesis, E2F targets, and G2M checkpoint (Figure 5(b)). The Riskscore was positively correlated with most oncogenic signaling pathways including hypoxia, MAPK, NFkB, TGFb, TNFa, TRAIL, and p53 in the TCGA-HCC cohort (Figure 5(c)). Additionally, the high-risk group had higher TMB, number of segments, fraction altered, proliferation, and transforming growth factor beta (TGF-beta)-response compared with the low-risk group (Figure 5(d)).

### 3.6. The Two Risk Groups Exhibited Different Immune Infiltration Landscapes

**Figure 6:**
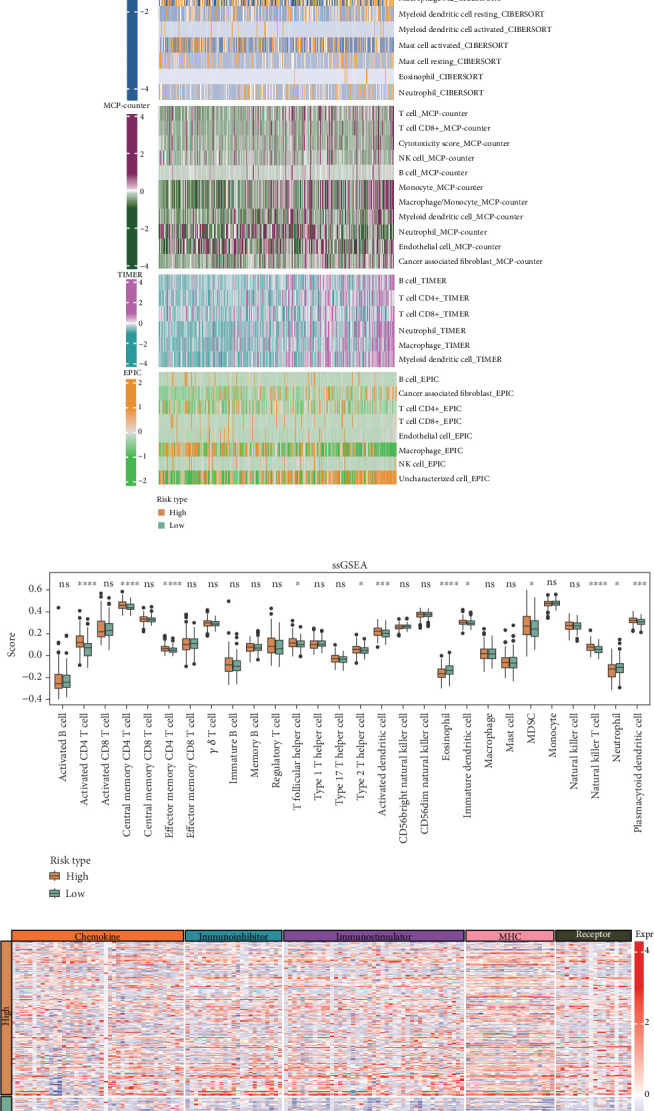
Analysis of immune cell infiltration between high- and low-risk groups in the TCGA-HCC cohort. (a) The infiltration levels of immune cells assessed by four algorithms, including CIBERSORT, MCP-counter, EPIC, and TIMER. (b) The infiltration scores of 28 immune cells calculated by ssGSEA. ⁣^∗∗∗∗^*p* < 0.0001; ⁣^∗∗∗^*p* < 0.001; ⁣^∗^*p* < 0.05; ns, not significant. (c) Expression levels of immunomodulator-related genes.

By integrating the results from CIBERSORT, MCP-counter, TIMER, EPIC, and ssGSEA algorithms, it was observed that the immune cell infiltration was significantly different in the risk groups. Notably, T cells such as Type 2 T helper cell, effector memory CD4 T cell, T follicular helper cell, natural killer T cell, activated CD4 T cell, and central memory CD4 T cell were more abundant in the high-risk group (Figures 6(a) and 6(b) and Supporting Information 1: Figure S1A–D). Moreover, the gene expression levels associated with immunomodulators (chemokine, immunoinhibitor, immunostimulator, MHC, and receptor) were notably elevated in the high-risk group in comparison to the low-risk group (Figure 6(c)).

### 3.7. Riskscore Model Could Accurately Predict the Immunotherapy Responses and Drug Sensitivities

**Figure 7:**
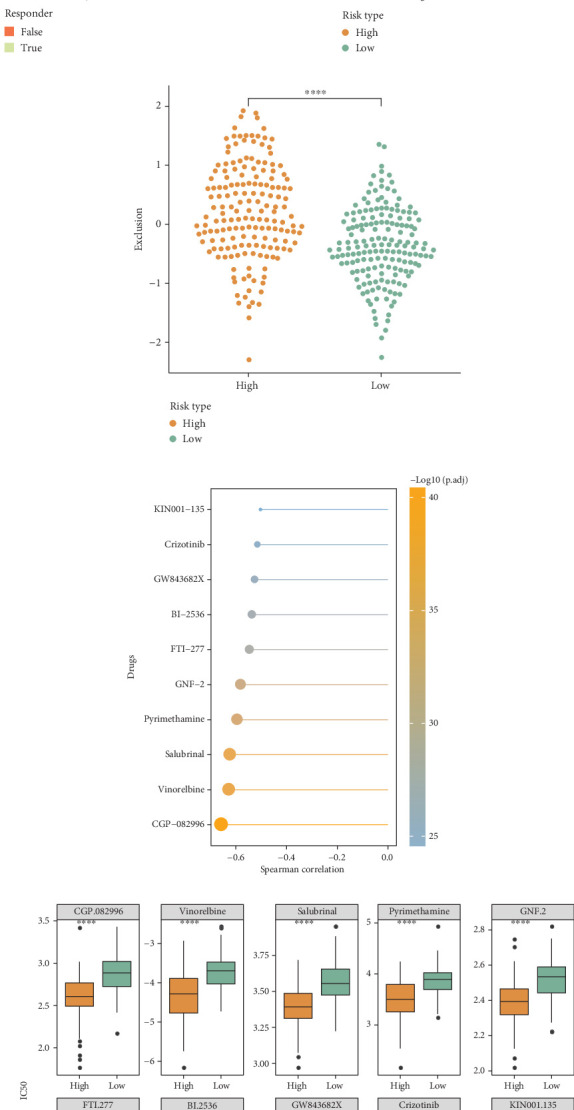
Assessment of immunotherapy response and drug sensitivity. (a) Immune response percentage between high- and low-risk groups in the TCGA-HCC cohort. (b) TIDE score between different risk groups. (c) Exclusion score between different risk groups. (d) Drugs correlated with Riskscore (|cor| > 0.5). (e) IC50 values between high- and low-risk groups. ⁣^∗∗∗∗^*p* < 0.0001.

The number of immunotherapy responders in the high-risk group (29.82%) was markedly lower than that in the low-risk group (42.11%) (Figure 7(a)). Compared to the low-risk group, the TIDE score and exclusion score of the high-risk group were markedly higher (Figures 7(b) and 7(c)). This suggested that high-risk HCC patients might have strong immune escape ability and poor immunotherapy efficacy. Furthermore, 10 chemotherapeutic drugs (such as vinorelbine, salubrinal, pyrimethamine, crizotinib) correlated with Riskscore were screened (|cor| > 0.5) (Figure 7(d)). The IC50 values of these 10 drugs in the high-risk group were notably lower than in the low-risk group (Figure 7(e)), indicating that high-risk HCC patients had higher sensitivities to the 10 drugs.

### 3.8. A Nomogram With Strong Prediction Ability Was Created

**Figure 8:**
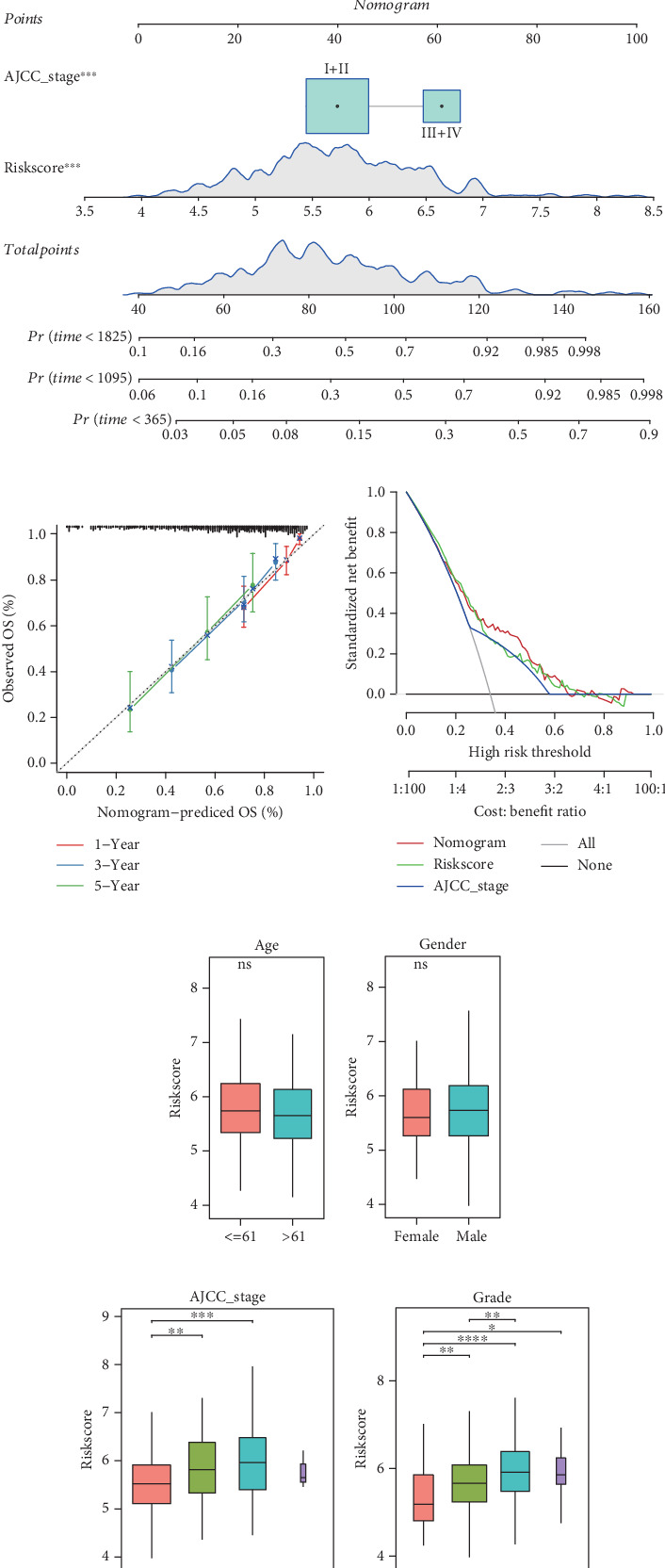
Establishment and verification of nomogram. (a, b) Univariate and multivariate analyses of Riskscore and clinical features. (c) Establishment of nomogram by combining Riskscore and AJCC stage. (d) Calibration curve of nomogram. (e) Decision curve of nomogram. (f–i) Correlation between Riskscore and clinical features. (j) C-index of nomogram, Riskscore, and clinical features. ⁣^∗∗∗∗^*p* < 0.0001; ⁣^∗∗∗^*p* < 0.001; ⁣^∗∗^*p* < 0.01; ⁣^∗^*p* < 0.05; ns, not significant.

Univariate and multivariate analysis showed that stage and Riskscore were the independent prognostic factors for HCC (Figures 8(a) and 8(b)). Then, the AJCC stage and Riskscore were integrated together to establish a nomogram (Figure 8(c)). The predicted calibration curves of the nomogram for 1, 3, and 5 years were close to the standard curves (Figure 8(d)). According to the DCA, the net benefit of the nomogram was higher than the other curves (Figure 8(e)), suggesting that the nomogram was dependable. In addition, no significant correlation between age, gender, and Riskscore was observed (Figures 8(f) and 8(g)). As the clinical AJCC stages (I, II, and III) and grades (G1, G2, G3, and G4) advanced, the Riskscore also increased (Figures 8(h) and 8(i)). Moreover, the C-index of the nomogram (0.72) and Riskscore (0.71) was higher than the other clinical indicators (Figure 8(j)). The above findings indicated that the nomogram exhibited strong survival prediction capability.

### 3.9. *CCT5* Silencing Reduced the HCC Cell Viability, Migration, and Invasion

**Figure 9:**
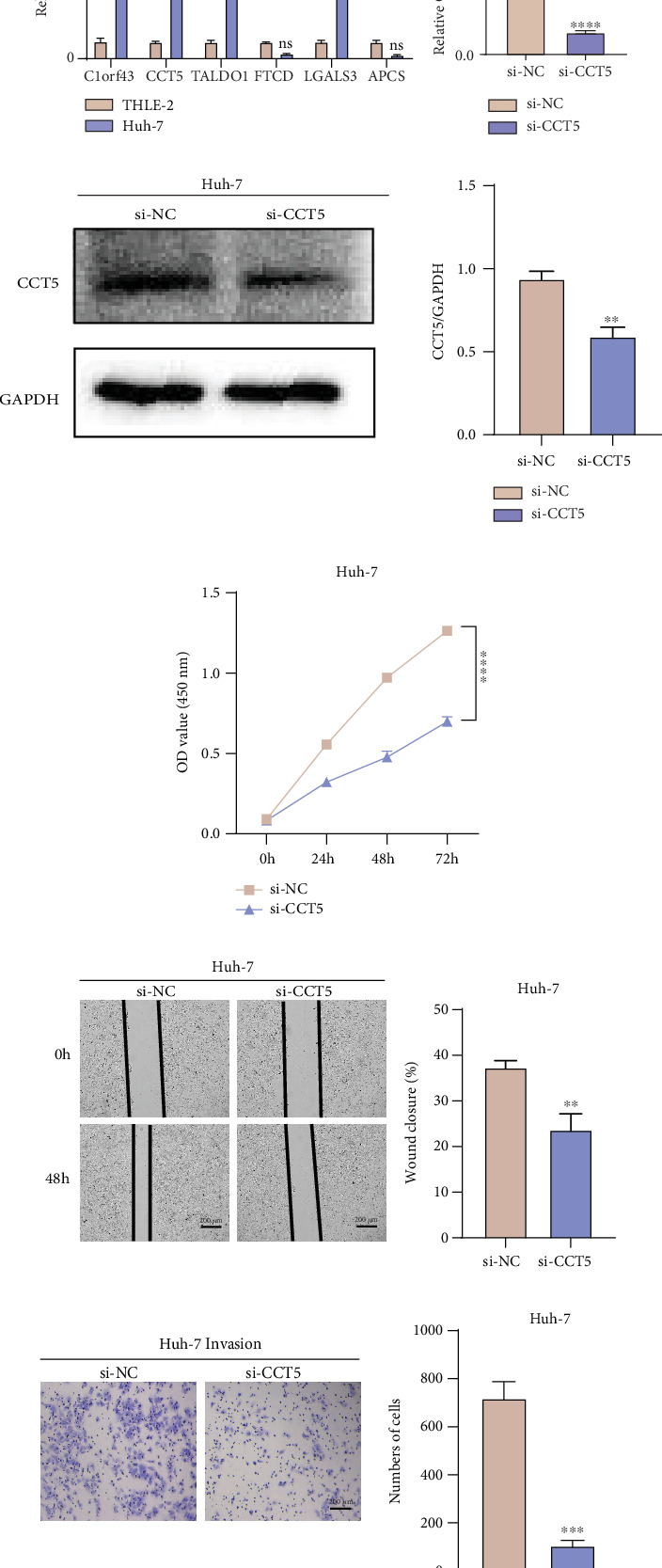
In vitro validation assays using HCC cells. (a) Relative mRNA expression levels of six prognostic LRGs in HCC cells Huh-7 and THLE-2 detected by qRT-PCR. (b, c) Based on qRT-PCR and Western blotting assays to assess the effect of silencing CCT5. (d) Impact of *CCT5* silencing on the cell viability of Huh-7 quantified via CCK-8 assay. (e) Effect of *CCT5* silencing on the cell migration of Huh-7 assessed by wound healing assay. (f) Impact of *CCT5* silencing on the cell invasion of Huh-7 via transwell assay. All data of three independent trials were expressed as mean ± standard deviation. ⁣^∗∗∗∗^*p* < 0.0001; ⁣^∗∗∗^*p* < 0.001; ⁣^∗∗^*p* < 0.01; ⁣^∗^*p* < 0.05.

To further examine the potential roles of LRGs in normal and tumor environments, we selected THLE-2 cells as normal hepatic stellate cells and Huh-7 cells as representative HCC cells for subsequent studies. Firstly, the qRT-PCR assay showed that *C1orf43*, *CCT5*, *TALDO1*, and *LGALS3* were observably upregulated in HCC cells Huh-7 in comparison to human hepatic stellate cells THLE-2 (Figure 9(a)). These results suggested that aberrant expressions of these genes functioned crucially in the onset and progression of HCC, showing their potential to be considered as the biomarkers for HCC. As a significantly high expression of *CCT5* in HCC cells was found, we further verified its efficiency after silencing using qRT-PCR and Western blot assays (Figures 9(b) and 9(c)). Further, the CCK-8 assay demonstrated that the silencing of *CCT5* reduced the viability of Huh-7 cells (Figure 9(d)). Wound healing and transwell assays revealed that silencing *CCT5* notably lowered the wound closure rate (Figure 9(e)) and the number of invasive Huh-7 cells (Figure 9(f)). These outcomes manifested that *CCT5* may be a critical risk factor facilitating tumor cell migration and invasion in the development of HCC.

## 4. Discussion

Lactylation, a newly discovered contributor to the epigenetic landscape, is often dysregulated in various malignancies [63]. The role of lactylation in the initiation of cancer and its progression has been gradually studied; however, the relationship between lactylation and HCC remains unclear. The present study screened six prognostic LRGs by LASSO regression analysis. Then, the Riskscore model with a strong performance in the prognostic assessment for HCC was developed. Utilizing the Riskscore, samples were separated into high- and low-risk groups. Specifically, the high-risk group showed higher T cell infiltration level, lower immunotherapy response rate, and higher sensitivity to chemotherapeutic drugs. Meanwhile, a nomogram with a high predictive accuracy was also developed.

Increasing studies have confirmed that LRGs can serve as a prognostic signature for multiple cancers [64], including HCC. For instance, Cai et al. [65] reported that the mRNA expressions of LRGs and lactylation levels were higher in HCC tissue than normal group and that upregulation of two key LRGs was closely related to a worse prognosis of HCC patients. Additionally, Cheng et al. [66] established and verified an eight-LRG signature for HCC, with a higher Riskscore indicating worse clinical outcomes. In our study, we also found the tumor group has a higher lactylation score than the normal group, and that the lactylation score was confirmed as an independent prognostic factor for HCC. Then, six prognostic LRGs were screened to construct a Riskscore model. Compared to the low-risk group, the high-risk patients had lower survival rate and a poorer prognosis. These findings indicated that the LRG signature could be considered as the biomarker for the clinical diagnosis and prognostic assessment of HCC.

The six prognostic LRGs in HCC contained two protective genes (*FTCD* and *APCS*) and four risk genes (*LGALS3*, *C1orf43*, *TALDO1*, and *CCT5*). *FTCD* encodes the enzyme required for the catabolism of histidine and tetrahydrofolate [67]. *FTCD* is expressed in most human tissues, with the highest expression level in the liver [68]. Studies reported that *FTCD* is involved in the HCC development and plays a potential role as a tumor-suppressive gene in HCC [69]. *APCS*, a member of the pentraxin family, encodes pathological serum amyloid P-component [70]. A wide involvement of *APCS* in innate immune response, tissue reshaping, and pathogenesis of inflammatory diseases shows its potential as a drug target for the therapy of amyloid diseases [71]. *APCS* is also a biomarker for indicating the prognostic outcomes for patients suffering from non-small cell lung cancer [72]. *LGALS3* is a galactose-specific lectin that plays an essential role in TME immunosuppression [73]. *LGALS3* is often upregulated in many malignant tumors [74]. It has been reported that *LGALS3* is correlated with a shorter survival time of HCC patients [75]. Currently, there were only few reports on the *C1orf43* in human diseases. In head and neck cancer, *C1orf43* has been identified as one of the 11 signature genes [76]. *TALDO1* is a critical enzyme in the pentose phosphate pathway, and the abnormal expression of *TALDO1* is relevant to the prognosis of various cancers [77]. Gene mutations of *TALDO1* can influence multiple organs with different clinical manifestations such as liver dysfunction and hepatosplenomegaly [78]. *TALDO1* has been identified as a prognostic gene for HCC [79]. *CCT5* belongs to a member of the chaperonin-containing TCP1 complex, and the protein-folding activity of *CCT5* is essential for cellular function [80]. The expression of *CCT5* is generally upregulated in cancers, which indicates a poor prognosis of patients [81]. Gain/loss-of-function tests showed that the abnormal expression of *CCT5* influences HCC cell proliferation, migration, and invasion [82]. Our present study discovered that *APCS* and *FTCD* were low-expressed but *C1orf43*, *CCT5*, *TALDO1*, and *LGALS3* were high-expressed in the high-risk group. In vitro assays revealed that silencing *CCT5* inhibited the viability, migration, and invasion of HCC cells Huh-7. Hence, the six LRGs may be promising targets for treating HCC in clinical practice.

An accurate evaluation of the TME can help individualize immunotherapy and identify patients suitable for immunotherapy [83]. In this study, the infiltration levels of T cells such as natural killer T cell, activated CD4 T cell, central memory CD4 T cell, T follicular helper cell, effector memory CD4 T cell, and Type 2 T helper cell were notably higher in the high-risk group. Previous studies reported that the enhancement of the immunosuppressive cells may confer high-risk patients a strong immunosuppressive TME, resulting in a poor outcome [84]. T cells exert a crucial role in the HCC progression. Immunotherapy mediated by natural killer T cells is promising in treating HCC with an inherent immunosuppressive TME [85]. The high-risk group showed lower immunotherapy responses and higher TIDE score, indicating that high-risk patients may have strong immune escape ability and poor immunotherapy efficacy [86]. Moreover, high-risk HCC patients were more sensitive to 10 drugs such as vinorelbine, salubrinal, pyrimethamine, and crizotinib, with a lower IC50. Vinorelbine is an antimicrotubule agent that can improve HCC tumor response to standard irradiation without increasing toxicity [87]. Salubrinal is a phosphatase inhibitor that can enhance pterostilbene-induced cell death in HCC via modulating endoplasmic reticulum stress pathway [88]. Pyrimethamine, a folic acid antagonist, induces apoptosis of HCC cells through inhibiting mitochondrial autophagy [89]. Crizotinib is an inhibitor of mesenchymal–epithelial transition factor that exhibits antitumor properties in various cancers [90]. These findings manifested that the Riskscore model developed based on the six LRGs had a great potential in guiding immunotherapy and targeted therapy of HCC.

Nevertheless, several limitations of our current study should be noticed. Firstly, the size of HCC samples obtained from TCGA and GEO databases was relatively small, and further validation of the research results using larger cohorts and clinical samples is required. Secondly, the prognostic significance of the six-LRG signature in HCC should be verified by combining clinical data. Finally, in vivo and in vitro experiments are needed to further examine the molecular mechanism of the six LRGs in the progression of HCC.

## 5. Conclusion

In conclusion, this study established a six-LRG Riskscore model that could accurately predict the prognosis of HCC patients. Different risk groups of HCC patients exhibited significant differences in immune cell infiltration, biological functions, clinical characteristics, immunotherapy responses, and drug sensitivities. The present study discovered six promising therapeutic targets and biomarkers for the management of HCC.

## Data Availability

The datasets generated and/or analyzed during the current study are available in the GSE112271 repository (https://www.ncbi.nlm.nih.gov/geo/query/acc.cgi?acc=GSE112271) and GSE43619 repository (https://www.ncbi.nlm.nih.gov/geo/query/acc.cgi?acc=GSE43619).

## Nomenclature

AJCC: American Joint Committee on Cancer
AUC: area under ROC curve
BP: biological process
CCK-8: cell counting kit-8
CI: confidence interval
CIBERSORT: cell-type identification by estimating relative subsets of RNA transcript
DCA: decision curve analysis
DEGs: differentially expressed genes
DMEM: Dulbecco's modified Eagle's medium
EPIC: estimating the proportions of immune and cancer cells
FBS: fetal bovine serum
FC: fold change
FPKM: fragments per kilobase of transcript per million fragments mapped reads
GEO: Gene Expression Omnibus
GO: Gene Ontology
GSEA: gene set enrichment analysis
HR: hazard ratio
IC50: half-maximal inhibitory concentration
K-M: Kaplan–Meier
LASSO: least absolute shrinkage and selection operator
HCC: hepatocellular carcinoma
LRG: lactylation-related gene
MCP-counter: microenvironment cell populations-counter
MHC: major histocompatibility complex
MSigDB: Molecular Signatures Database
OS: overall survival
PCA: principal component analysis
PFS: progression-free survival
qRT-PCR: quantitative real-time polymerase chain reaction
ROC: receiver operating characteristic
scRNA-seq: single-cell RNA sequencing
si: small interfering
ssGSEA: single-sample gene set enrichment analysis
TCGA: The Cancer Genome Atlas
TIDE: tumor immune dysfunction and exclusion
TIMER: tumor immune estimation resource
TILs: tumor-infiltrating lymphocytes
TMB: tumor mutational burden
TME: tumor microenvironment
TPM: transcripts per million
UMAP: uniform manifold approximation and projection

## Supporting Information

Supporting Information 1 Figure S1: The infiltration levels of immune cells between high- and low-risk groups in the TCGA-HCC cohort. (a) The infiltration scores of 22 immune cells assessed by the CIBERSORT algorithm. (b) The infiltration scores of six immune cells evaluated by the TIMER algorithm. (c) The infiltration scores of eight immune cells calculated by the EPIC algorithm. (d) The infiltration scores of 10 immune cells calculated by the MCP-counter algorithm. ⁣^∗∗∗∗^*p* < 0.0001; ⁣^∗∗^*p* < 0.01; ⁣^∗^*p* < 0.05; ns, not significant.

Supporting Information 2 Table S1: Primer sequences used in this study.
