# Supplementary figures and images for: Establishment of a Lactylation-Related Gene Signature for Hepatocellular Carcinoma Applying Bulk and Single-Cell RNA Sequencing Analysis
Source: Int J Genomics. 2025 Feb 14;2025:3547543. doi: 10.1155/ijog/3547543 (PMC11845269; doi:10.1155/ijog/3547543)

**A**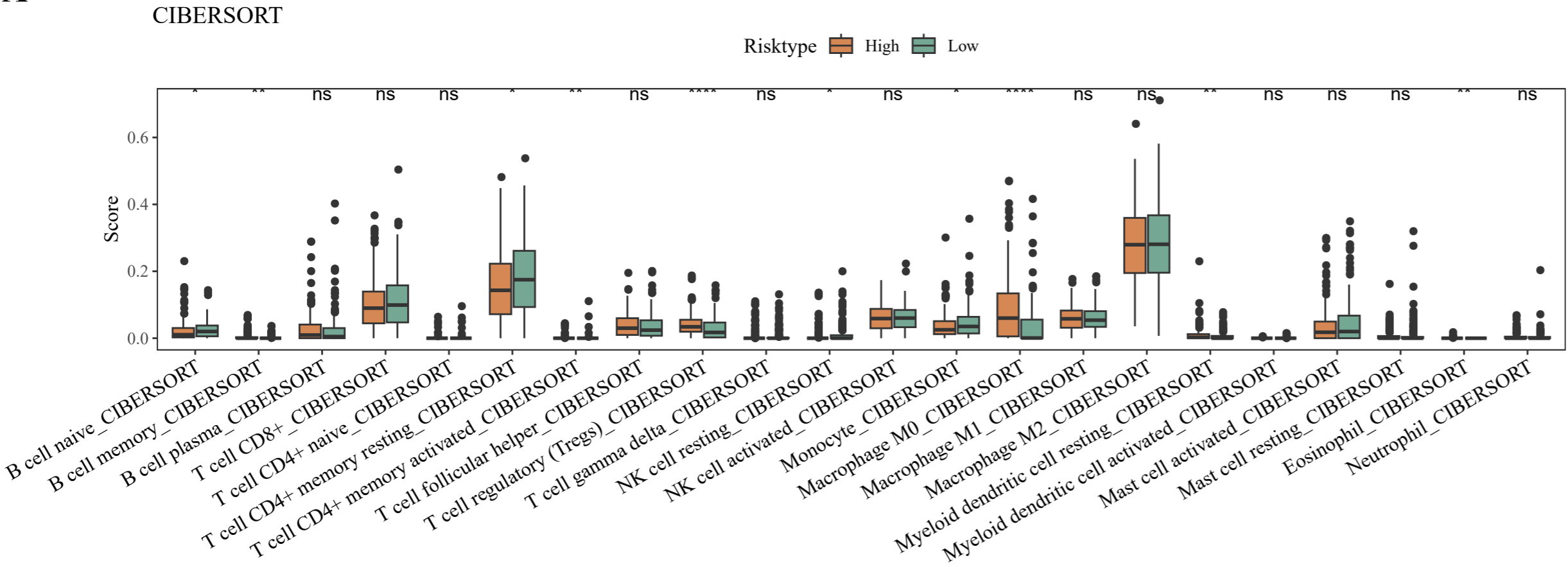**B**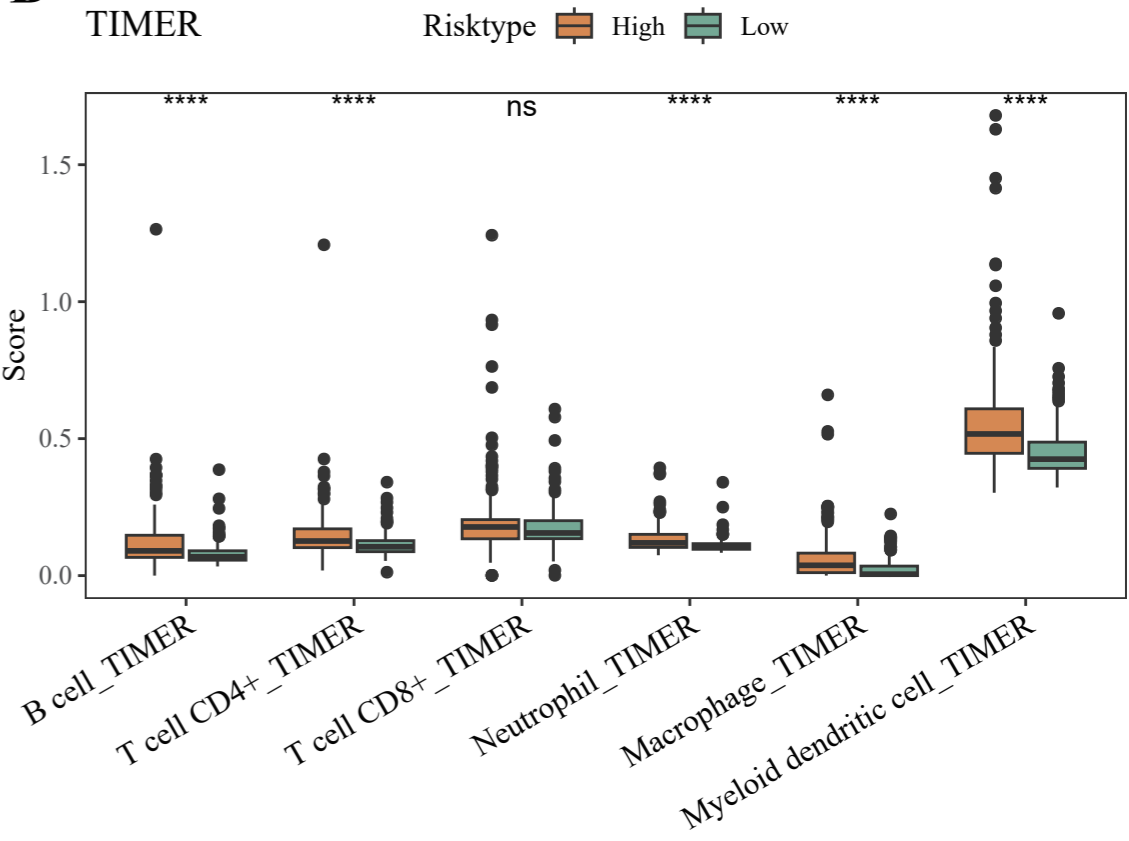**C**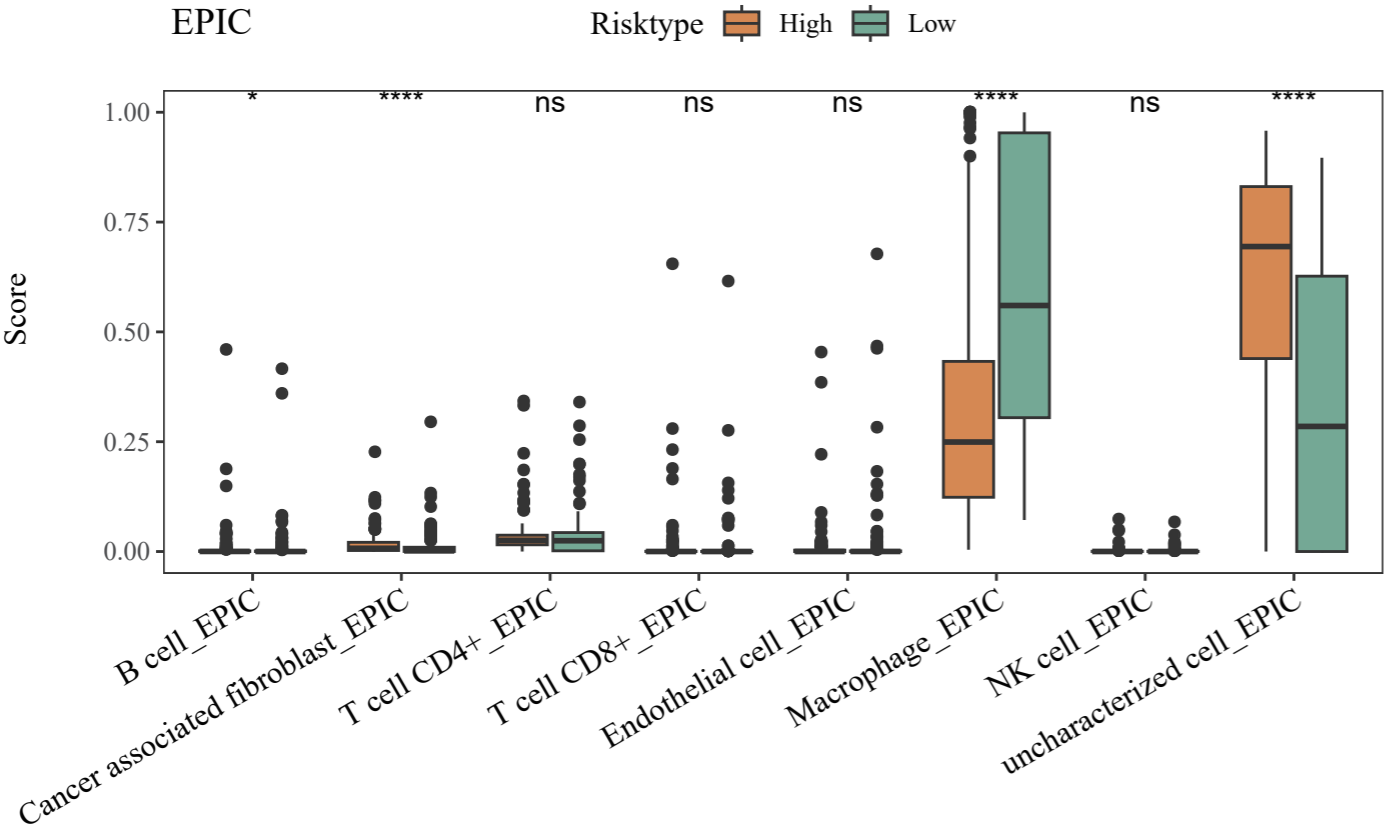**D**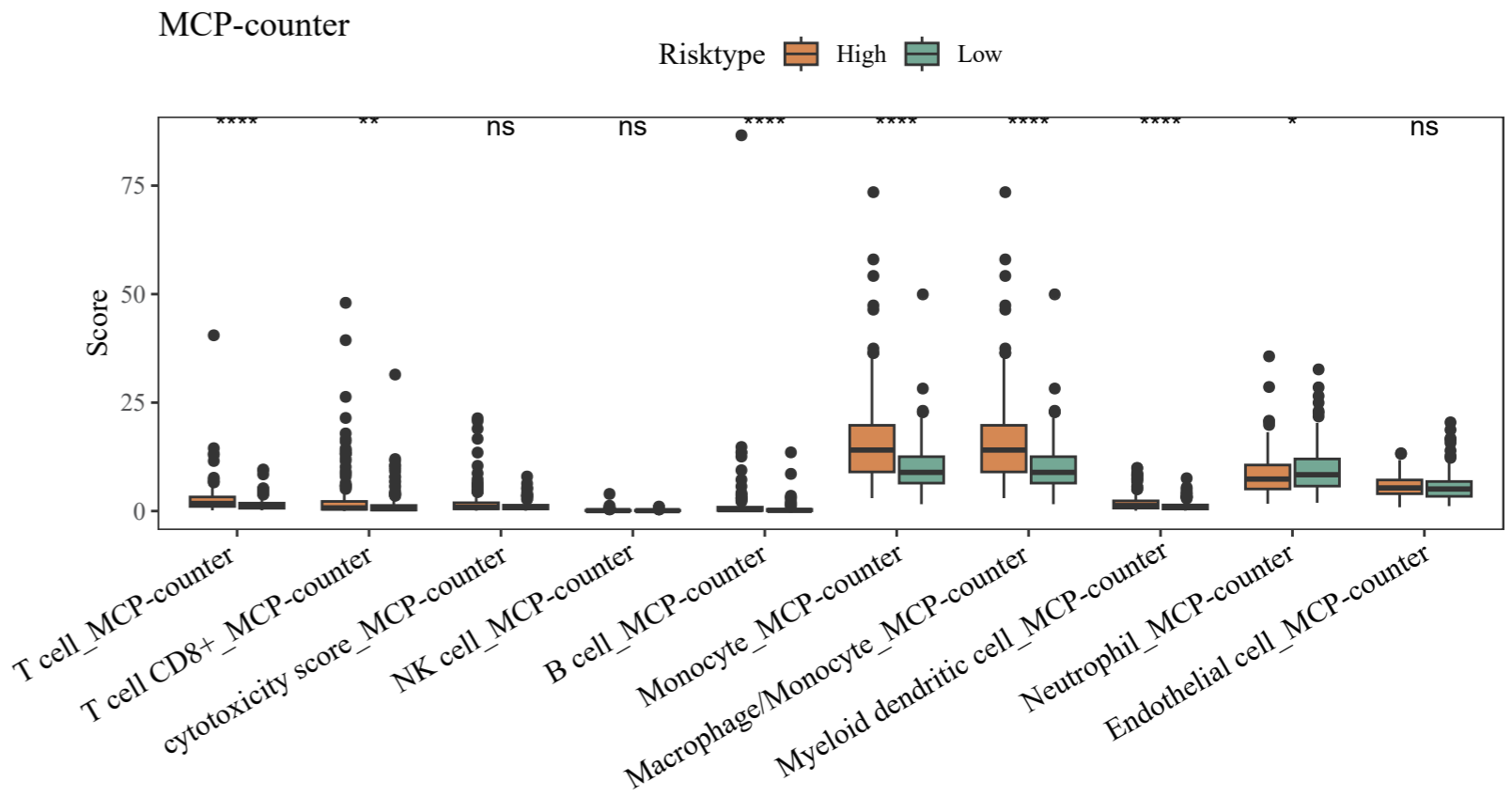

Supplement: Supporting Information 1 — Figure S1: The infiltration levels of immune cells between high- and low-risk groups in the TCGA-HCC cohort. (a) The infiltration scores of 22 immune cells assessed by the CIBERSORT algorithm. (b) The infiltration scores of six immune cells evaluated by the TIMER algorithm. (c) The infiltration scores of eight immune cells calculated by the EPIC algorithm. (d) The infiltration scores of 10 immune cells calculated by the MCP-counter algorithm. ⁣∗∗∗∗p < 0.0001; ⁣∗∗p < 0.01; ⁣∗p < 0.05; ns, not significant. [file 3547543.f1.pdf]
