# Supplementary material for: Establishment of a Lactylation-Related Gene Signature for Hepatocellular Carcinoma Applying Bulk and Single-Cell RNA Sequencing Analysis
Source: Int J Genomics. 2025 Feb 14;2025:3547543. doi: 10.1155/ijog/3547543 (PMC11845269; doi:10.1155/ijog/3547543)
Supplement: Supporting Information 2 — Table S1: Primer sequences used in this study. [file 3547543.f2.docx]

**Supplementary Table 1. Primers** **sequences used in this study**

| Gene | Primers (5’-3’) |
| --- | --- |
| *C1orf43* | Forward: GTATAGGATGAAAGCTCTGG  Reverse: CTCCTGATAGCGTAGGTACT |
| *CCT5* | Forward: CGTCTTATGGGACTTGAG  Reverse: CTAGGTTAGCACCAGTCTCT |
| *TALDO1* | Forward: GTTGTTTGGAGCAGAAATAC  Reverse: CATCTTTATCAAAGGAGAGC |
| *FTCD* | Forward: GAGAAGGAGAACCTCTTCAT  Reverse: GTGGATCTGGTCCTTAAAT |
| *LGALS3* | Forward: GAGAACAACAGGAGAGTCAT  Reverse: GATGATTGTACTGCAACAAG |
| *APCS* | Forward: TAGTGATCTCTCTCGTGCCTAC  Reverse: GCCGGGAACTTTTCGATAACTT |
| *GAPDH* | Forward: GGAGCGAGATCCCTCCAAAAT  Reverse: GGCTGTTGTCATACTTCTCATGG |
